# Modeling, Simulation and Data Processing for Additive Manufacturing

**DOI:** 10.3390/ma14247755

**Published:** 2021-12-15

**Authors:** Mika Salmi

**Affiliations:** Department of Mechanical Engineering, Aalto University, 02150 Espoo, Finland; mika.salmi@aalto.fi

Additive manufacturing or, more commonly, 3D printing is one of the fundamental elements of Industry 4.0. and the fourth industrial revolution. It has shown its potential example in medical and dentistry, automotive, aerospace, and spare parts [1,2,3,4,5]. Personal manufacturing, complex and optimized parts, short series manufacturing and local on-demand manufacturing are some of the current benefits. Development of process and materials in terms of speed, costs and availability open new business cases all the time. Most of the research has focused on material and AM process development or effort to utilize existing materials and processes for industrial applications. However, improving the understanding and simulation of materials and AM process and understanding the effect of different steps in the AM workflow can increase the performance even more. The best way of benefit of AM is to understand all the steps related to that—from the design and simulation to additive manufacturing and post-processing ending the actual application.

This Special Issue consists of 10 original full-length articles on modeling, simulation and data processing for AM. Li et al. [6] studied finite element modeling of a novel lattice bimetallic composite comprising 316L stainless steel and a functional dissolvable aluminum alloy. Samples were fabricated and characterized, and experimental, finite element analysis (FEA) and digital image correlation (DIC) results were compared. The dissolvable aluminum showed higher Young’s modulus, yield stress, and ultimate stress than the lattice and composite, but less elongation. Moreover, they demonstrated FEA and DIC efficient methods to simulate, analyze, and verify the experimental results. Sikan et al. [7] developed a finite element model for electron beam additive manufacturing of Ti-6Al-4V to understand the metallurgical and mechanical aspects of the process. Thin wall plates of 3 mm thickness were fabricated to validate the simulation results and ensure the reliability of the developed model. The thermal predictions of the model, when validated experimentally, gave a low average error of 3.7%. The model proved to be highly successful for predicting the cooling rates, grain morphology, and microstructure. The maximum deviations observed in the mechanical predictions of the model were as low as 100 MPa in residual stresses and 0.05 mm in distortion.

Singh et al. [8] used the finite element method to understand the influences of laser power and scanning speed on the heat flow and melt-pool dimensions in the powder bed fusion process for titanium and Inconel 718. A transient 3D finite-element model was developed to perform a quantitative comparative study to examine the temperature distribution and disparities in melt-pool behaviors under similar processing conditions. The temperature and melt-pool increase as laser power moves in the same layer and when new layers are added. The same is observed when the laser power increases. The opposite is observed for increasing scanning speed while keeping other parameters constant. Luo et al. [9] established a multi-layer and multi-track finite element model of 24CrNiMo alloy steel for powder bed fusion. The distribution and evolution of temperature and stress fields and the influence of process parameters on them were systematically studied. The results showed that the peak temperature increases from 2153 °C to 3105 °C, and the residual stress increases from 335 MPa to 364 MPa by increasing laser power from 200 W to 300 W; the peak temperature decreases from 2905 °C to 2405 °C, and the residual stress increases from 327 MPa to 363 MPa at scanning speeds from 150 mm/s to 250 mm/s; the peak temperature increases from 2621 °C to 2914 °C and the residual stress decreases from 354 MPa to 300 MPa at preheating temperatures of 25 °C and 400 °C, respectively.

Rupal et al. [10] systematically designed a benchmark for geometric tolerance characterization that can present tolerances in three principal planar directions. The benchmark was simulated using the finite element method, made with the LPBF process from stainless steel (316L), and the geometric tolerances were characterized. The effect of base plate removal on the geometric tolerances was quantified. Simulation and experimental results were compared to understand tolerance variations using different process parameters such as base plate removal, orientation, and size. Budzik et al. [11] presented a quality control methodology for additive manufacturing products made of polymer materials. The methodology varies depending on the intended use. Depending on the use of the models, the quality control process is divided into three stages: data control, manufacturing control, and post-processing control. When selecting materials, the 3D printing process and measurement methods, the purpose of the model and economic aspects should be taken into account. All products do not require high accuracy and durability.

Kusoglu et al. [12] studied nanoparticle additivation effects on LPBF of metals and polymers. They formed a theoretical concept for an inter-laboratory study design considering the process chain, including research data management. Macioł et al. [13] compared automatically detected precipitates in LPBF of Inconel 625 with a combination of the complementary electron techniques such as the chemical composition performed by EDS. Image processing methods and statistical tools were applied to maximize information gain from data with a low signal-to-noise ratio, keeping human interactions on a minimal level. The proposed algorithm allowed for the automatic detection of precipitates.

Kuschmitz et al. [14] used machine-learning techniques to compute acoustic material parameters from the material’s micro-scale geometry for the additively manufactured porous sound absorbers. Laboratory measurements data of the flow resistivity and absorption coefficient were used to train two different machine learning models, an artificial neural network and a k-nearest neighbor approach. Both models could predict acoustic parameters from the specimen’s micro-scale with reasonable accuracy. Salmi [15] reviewed additive manufacturing processes and materials in medical applications and process workflow for different uses. Based on the findings, directed energy deposition is rarely utilized in implants and sheet lamination for medical models or phantoms. Powder bed fusion, material extrusion and VAT photopolymerization are utilized in all categories. Material jetting is not used for implants and biomanufacturing, and binder jetting is not utilized for tools, instruments and parts for medical devices. The most common materials are thermoplastics, photopolymers and metals such as titanium alloys.

## Data Availability

Not applicable.
